# Ankylosing spondylitis functional and activity indices in clinical practice

**Published:** 2014-03-25

**Authors:** C Popescu, M Trandafir, AM Bădică, F Morar, D Predeţeanu

**Affiliations:** * "Sfânta Maria" Clinical Hospital, Bucharest; ** "Carol Davila" University of Medicine and Pharmacy, Department of Internal Medicine and Rheumatology, Bucharest

**Keywords:** ankylosing spondylitis, BASFI, BASDAI, ASDAS

## Abstract

Background: Clinicians have at hand several indices to evaluate disease activity and functionality in ankylosing spondylitis (AS), in order to evaluate the prognostic and the treatment of AS patients.

Objectives: to examine the relationship between functional and activity scores in AS; to note whether disease activity is associated with any clinical or laboratory variables.

Methods: the study included AS patients, classified according to the revised New York criteria; data recorded: demographics, disease duration, type of articular involvement, HLA B27 presence, history of uveitis, calculation of BASFI, BASDAI and ASDASCRP, quantification of inflammation markers.

Results: 50 AS patients; ASDASCRP correlated significantly (p < 0.001) with BASFI (r = 811), BASDAI (r = 0.810) and with erythrocyte sedimentation rate (ESR; r = 0.505); HLA B27 positive patients had a median BASDAI 5 times higher than HLA B27 negative patients (p = 0.033); compared with patients with strictly axial disease form, patients with axial and peripheral disease had a median ESR 3 times higher (p = 0.042) and a median BASDAI 2 times higher (p = 0.050).

Conclusions: functional and activity AS indices are strongly correlated in assessing disease severity; inflammation and HLA B27 can predict the high value of these indices; axial and peripheral disease pattern is associated with higher disease activity.

## Introduction

Ankylosing spondylitis (AS) is the prototypical nosological entity of the spondilarthritis group, which encompasses reactive arthritis, psoriatic arthritis, inflammatory bowel disease etc. [**1**]. AS is pathogenically characterized by a chronic inflammatory state of unknown etiology, which mainly affects the spine and the sacroiliac joints, but also extra-spinal (e.g. peripheral joints) and extra-articular (e.g. anterior pole of the eye) areas. The typical clinical aspects of the disease are inflammatory chronic back pain, radiographic sacroiliitis and the presence of human leukocyte antigen (HLA) B27, all of which are very useful diagnostic tools. Disease activity leads to severe anatomical deformity (e.g. kyphosis), to various degrees of functional impairment (e.g. the limitation of lumbar flexion) and to secondary psychological repercussions (e.g. anxiety and depression related to chronic pain), that severely alter the patients’ quality of life and lead to higher social costs, proportionally with disease duration [**2,3**]. To prevent such negative outcomes, rheumatologists must intervene in two crucial moments in the natural evolution of AS: on one hand, to diagnose early the disease and on the other hand to quantify and control its activity. In clinical practice, functional evaluation is done with the Bath Ankylosing Spondylitis Functional Index (BASFI),[**4**] and disease activity is quantified with two score: an earlier one, containing only subjective clinical elements, Bath Ankylosing Spondylitis Disease Activity Index (BASDAI); [**5**] a new one, containing both subjective clinical elements and objective laboratory measures, Ankylosing Spondylitis Disease Activity Score (ASDAS), which allows to classify an AS patients as having an inactive disease (ASDAS < 1.3), a moderate disease activity (ASDAS < 2.1), a high disease activity (ASDAS = 2.1 - 3.5) or a very high disease activity (ASDAS > 3.5) [**6,7**]. The above-mentioned indices offer a quantifiable expression of disease activity, but also a target and a monitoring variable of treatment. In this context, the present study aims to examine the relationship between functional and disease activity AS scores and to observe any clinical or laboratory element associated with higher AS activity.

## Materials and methods

The analyzed population sample included 57 adults known to have AS, recruited randomly, by the order in which they requested medical assistance. Each participant in the study gave informed consent and the protocol was approved by the local ethics committee. Two rheumatologists evaluated the patients, including history of disease, clinical examination, filling in BASFI, BASDAI and ASDAS forms, reviewing and supplementing sacroiliac joints imagistic investigations, dosing inflammatory markers: erythrocyte sedimentation rate (ESR) was determined using the Westergern method (normal values < 20-30 mm/h according to sex and age); C-reactive protein (CRP) levels were determined with an immunonephelometric assay (normal values < 5 mg/L). Normally distributed data were reported as means with standard deviations, while non-normally distributed data were reported as medians with range and qualitative data were reported in absolute value with percent of total. Differences were evaluated using non-parametric tests: binomial and χ2 tests (or Fisher’s exact test where appropriate) for nominal data; Mann-Whitney U and Kruskal Wallis tests for scale data. Correlation was established computing Spearman’s coefficients. Where the data allowed, simple linear regression was performed. Specificity and sensitivity of the indices were assessed with receiver operating characteristics (ROC) curves, assuming a 50% pretest probability and a cost ratio of 1. All tests were two-sided, were considered significant if p ≤ 0.05 and were done using SPSS Statistics v.17.0.1 for Windows (SPSS Inc., Chicago, U.S.A., 2008).

## Results

Of the 57 subjects included in the study, only 50 fulfilled the revised New York classification criteria for AS, [**8**] while 7 fulfilled the ASAS classification criteria for axial and peripheral spondylarthitis [9,10]. Table 1 summarizes the general characteristics of the 50 AS patients retained for analysis. This sample contained mostly men with active mixed (axial and peripheral) involvement, HLA B27 positive, treated with anti-TNFα agents (infliximab, adalimumab, etanercept, golimumab). 

**Table 1:**
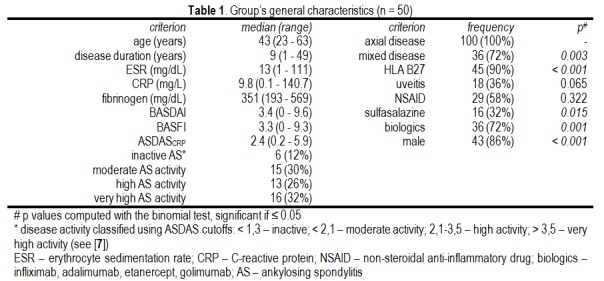
Group’s general characteristics (n = 50)

**Fig. 1:**
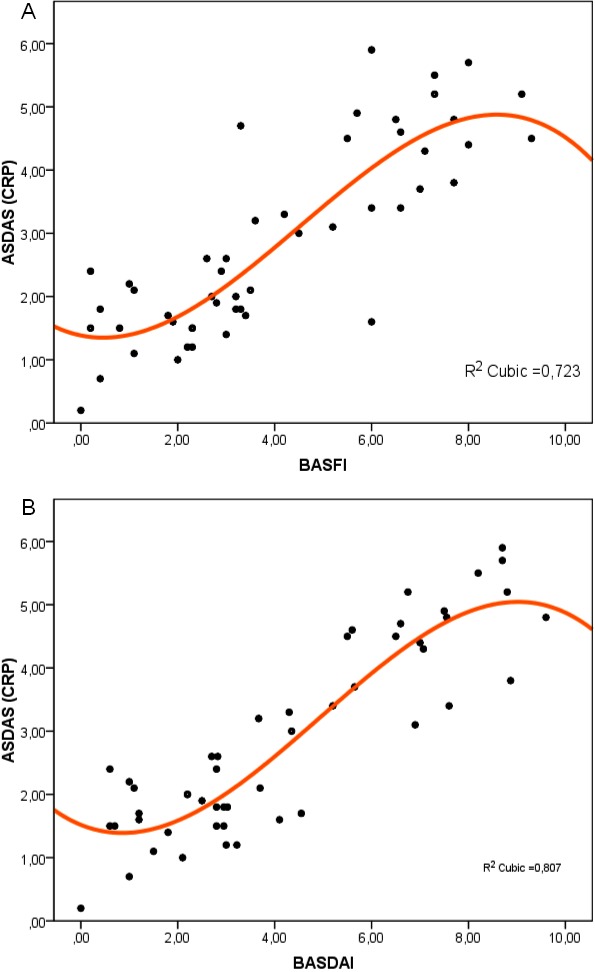
Scatter plots with ASDASCRP correlation with BASFI (panel A) and BASDAI (panel B), using cubically fitted trend lines

The functional index and the two activity indices were significantly correlated, both among themselves, but also with age on examination and inflammatory markers (Table 2, Fig. 1). The functional index (BASFI) correlated stronger with the subjective activity index (BASDAI) than with the objective activity index (ASDASCRP). 

**Table 2:**
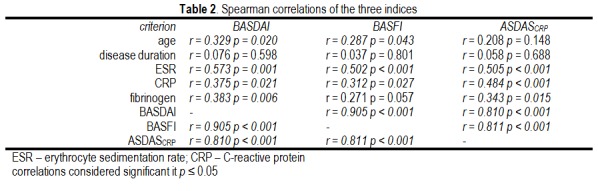
Spearman correlations of the three indices

 These continuous variables were good predictors of functional impairment and disease activity, contributing significantly to their respective values (**Table 3**). For example, for each year of age, BASDAI increased with 1.5% and BASFI increased with 1.6%. Similarly, for each 10 mm/h increment in ESR, BASDAI increased with 12.7%, BASFI increased with 10.2% and ASDASCRP increased with 11.4%. 

In general, the continuous variable, including functional and activity indices, did not differ significantly between the subgroups divided by the presence or the absence of mixed disease. There were two notable exceptions: mixed disease AS patients had a higher median ESR than strictly axial disease patients (25 mm/h; 8 mm/h; p = 0.042); mixed disease patients had a higher median BASDAI than strictly axial disease patients (4.2; 2.35; p = 0.050). HLA B27 patients had a median BASDAI significantly higher than patients lacking this antigen (3.7; 0.7; p = 0.033), with no differences regarding BASFI (p = 0.086) or ASDASCRP (p = 0.058). Compared to patients not receiving non-steroidal anti-inflammatory drug (NSAIDs), patients who received this treatment had significantly higher median ESR (31 mm/h; 8 mm/h; p = 0.008) and functional and activity indices (BASFI: 5.2, 2.8, p = 0.035; BASDAI: 5.2, 2.9, p = 0.042; ASDASCRP: 3.3, 1.8, p = 0.011). 

**Table 3:**
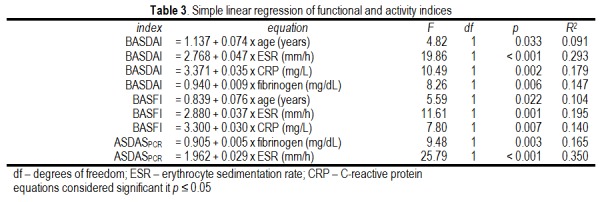
Simple linear regression of functional and activity indices

 Compared to patients not receiving sulfasalazine (SSZ), patients on this drug had significantly higher median CRP (22.7 mg/L, 6.6 mg/L, p = 0.012), BASDAI (6.7, 2.8, p = 0.008) and ASDASCRP (4, 2, p = 0.005), with no significant difference regarding BASFI (p = 0.058). SSZ indications of treatment reflected in the 15 times higher frequency with which mixed disease patients received it compared to strictly axial disease patients (30%, respectively 2%; p = 0.021). All the patients without SSZ treatment (n = 36) were male (p < 0.001) and among them 30 received anti-TNFα agents (83.3%), while 6 did not receive any disease-modifying drug (16.7%; p = 0.011). Compared with patients not treated with anti-TNFα biologics, patients on this treatment had significantly lower median BASDAI values (3, 6.75, p = 0.020), with no significant difference regarding BASFI (p = 0.077) and ASDASCRP (p = 0.219). Mixed disease pattern was associated with a higher frequency of anti-TNFα treatment: of the 36 patients receiving TNFα blockers, 29 had mixed disease and 7 had axial disease only (p = 0.042). The only significant difference between sex subgroups was a higher median disease duration in males (10 years, 4 years, p = 0.013) [**11**]. Patients with a history of uveitis were not significantly different from patients without uveitis. 

**Fig. 2:**
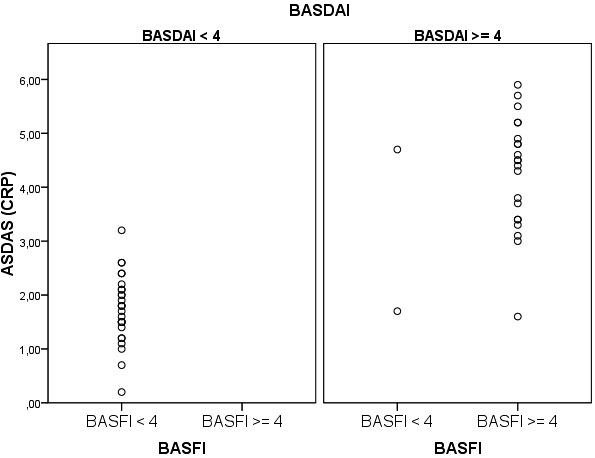
Distribution of values according to BASDAI and BASFI subgroups

 Regarding disease activity, the inflammatory markers and the functional and activity indices (BASFI, BASDAI) followed the ASDASCRP hierarchy (inactive, moderate activity etc.), in the sense that each AS activity subgroup had median ESR (p = 0.005), CRP (p = 0.012), fibrinogen (p = 0.004), BASDAI (p < 0.001) and BASFI (p < 0.001) significantly higher than the inferior subgroup and lower than the superior subgroup (Kruskal Wallis test). Compared to patients with BASDAI or BASFI < 4, patients with BASDAI/BASFI ≥ 4 had a significantly higher prevalence of inflammation (**Table 4,Fig. 2**). 

**Table 4:**
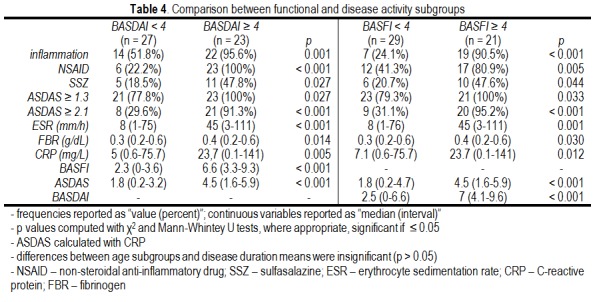
Comparison between functional and disease activity subgroups

There were 2 possible errors given by a conflict between activity scores: one patient with BASDAI ≥ 4, BASFI < 4 and ASDASCRP = 1.7; and another with BASDAI ≥ 4, BASFI ≥ 4 and ASDASCRP = 1.6. A third anomaly was the case of a patient with BASDAI ≥ 4, BASFI < 4 and ASDASCRP = 4.7, who had a disease duration of 12 months (which may account for low BASFI) and high inflammatory markers. 

 ASDASCRP and BASFI were very good tests for identifying patients with BASDAI ≥ 4 (area under ROC curve = 0.9485, 95% CI: 0.881-1, respectively 0.9928, 95% CI: 0.978-1). BASFI, with a > 3.9 cutoff and 91.3% sensitivity and 100% specificity, was better that ASDASCRP (> 2.8 cutoff, 91.3% sensitivity, 96.3% specificity) for identifying these patients. Analogous, ASDASCRP and BASDAI were very good tests for identifying patients with BASFI ≥ 4 (area under ROC curve = 0.9483, 95% CI: 0.880-1, respectively 0.9819, 95% CI: 0.951-1). Again, BASDAI, with a > 3.9 cutoff and 100% sensitivity and 93.1% specificity, was better that ASDASCRP (> 2.8 cutoff, 95.2% sensitivity, 93.1% specificity) for identifying these patients. 

## Discussion

This study reports a high degree of correlation between the functional index (BASFI) and the clinical activity indices (BASDAI, ASDASCRP) [**12**]. In other words, using either one of these indices will equivalently quantify disease severity at a certain examination time. Although the statistical tests used demonstrated only the degree of association between the three indices, it is obvious that the determining factor behind this association is the AS diagnosis itself, a fact confirmed by their correlation with inflammatory markers. The strength of this correlation was enough to allow inflammatory markers to predict the functional and activity status of the disease. It is a previously noted observation which led to the insertion of ESR and CRP in clinical disease activity index, increasing the objectiveness of the evaluation process by the emergence of the ASDAS [**6**]. Our data confirmed the validity of the ASDAS cut-offs used to classify AS. For example, a patient with moderate disease activity (ASDAS = 1.3 - 2.1) had significantly higher titers of inflammatory markers (ESR, CRP, fibrinogen) and significantly higher subjective indices (BASDAI, BASFI) when compared to a patient with inactive disease (ASDAS < 1.3). Besides the advantage of ASDAS that in incorporates a laboratory measure, it also brings the benefit of a hierarchal classification of disease activity, very useful in judging responses to therapy. Functional impairment and high disease activity (BASDAI/BASFI ≥ 4) were paralleled by ASDASCRP values and were associated with a higher frequency of inflammation and NSAID and SSZ treatment. In fact, ASDASCRP was a very good test for detecting highly active AS (BASDAI/BASFI ≥ 4), with sensitivity and specificity higher than 91%. For example, if a patient with BASDAI ≥ 4 has an ASDASCRP > 2.8, that patient surely has a very active disease (96.3% specificity), which needs intense therapeutic management. Analogous, if a patient with BASFI ≥ 4 has an ASDASCRP < 2.8, his or hers BASFI must be recalculated (95.2% sensitivity) for it is highly probable that an error occurred pertaining either to the patient or to the calculation itself. Therefore, it is advisable to routinely calculate ASDAS in patients with BASDAI/BASFI ≥ 4, for a more objective disease status. 

 In spite of the fact that BASDAI and BASFI do not integrate the age of the patient, its influence on these subjective functional and activity indices can be explained by the presence of primary and secondary osteoarthritis, which may lead to their nonspecific increase. One expects that limb AS arthritis and secondary osteoarthritis will affect more profoundly the patient’s functional status and quality of life. Indeed, our group had a high prevalence of peripheral involvement and these patients had a significantly higher median ESR and BASDAI when compared to strictly axial disease pattern. 

 HLA B27 is a strong genetic risk factor for AS and its presence is associated with a higher prevalence of uveitis and cardiac involvement [13,14]. In our group, 18 patients had a history of uveitis (36%), of which 17 carried HLA B27 (94.4%). However, of the 45 patients carrying HLA B27 (90%), only 17 had a history of uveitis (37.8%), which made the difference not statistically significant (p > 0.05). Instead, as other authors have observed, [**15**] the HLA B27 subgroup had a more active disease (median BASDAI vales in our sample) when compared to patients without HLA B27 the antigen. This difference cannot by accounted for by the uveitis’ effect on quality of life, since none of our patients had uveitis at the time of inclusion in the study, nor did they have significantly impairing sequelae on vision, but only a history of this extra-articular involvement, confirmed by ophthalmologists. The difference may be explained by a higher prevalence of extra-articular involvement in the HLA B27 subgroup, [**15**] data which were not recorded by the present study. 

 Unexpectedly, patients who were receiving NSAID and SSZ treatment at the time of inclusion in the study had significantly higher inflammation markers and functional and activity indices. This observation does not reflect disease duration, since the NSAID and SSZ subgroups were not different in this regard, but it possibly reflects treatment duration and the concurrent administration of anti-TNFα agents. Of the 36 patients receiving TNFα blockers (72%), only 6 had concurrent SSZ treatment (16.7%), while 30 did not (83.3%, p = 0.011). Therefore, the higher disease activity of NSAID and SSZ patients may indicate the need of anti-TNFα blockade. 

 Our data underlined the severity of peripheral and axial (mixed) involvement in AS. Thus, patients with mixed disease had higher inflammation markers, BASDAI and anti-TNFα treatment frequency compared to patients without peripheral involvement. It is possible that the small sample size (50) and relatively high prevalence of peripheral involvement in our group (72%, p = 0.003) generated these differences. That is why studies on bigger samples, with equivalent prevalence of mixed and strictly axial involvement are needed to confirm these results.

## Conclusions

 The functional index (BASFI) and the two activity indices (BASDAI, ASDAS) are strongly inter-correlated when assessing AS severity, both by their absolute values and by the ASDAS hierarchy. ASDAS is a very specific and sensitive test for detecting high activity low functionality AS patients. The inflammatory markers predict the three indices’ values, even in the case of those that do not integrate ESR or CRP (BASDAI, BASFI). Other significant predictors of disease activity are the presence of HLA B27 and the mixed (peripheral and axial) disease pattern, which require treatment with anti-TNFα agents. 

 Conflicts of interest 

 None declared. 

 Acknowledgements 

 The author would like the thank physicians from “Sfanta Maria" Clinical Hospital, Bucharest, Clinic of Internal Medicine and Rheumatology, for providing patients. 
